# Using artificial neural networks to explain the attraction of jewel beetles (Coleoptera: Buprestidae) to colored traps

**DOI:** 10.1111/1744-7917.13496

**Published:** 2025-01-16

**Authors:** Roger D. Santer, Otar Akanyeti

**Affiliations:** ^1^ Department of Life Sciences Aberystwyth University Aberystwyth Wales UK; ^2^ Department of Computer Science Aberystwyth University Aberystwyth Wales UK

**Keywords:** Buprestid, emerald ash borer, flathead oak borer, multifunnel trap, prism trap, trap color

## Abstract

Jewel beetles pose significant threats to forestry, and effective traps are needed to monitor and manage them. Green traps often catch more beetles, but purple traps catch a greater proportion of females. Understanding the function and mechanism of this behavior can provide a rationale for trap optimization. Jewel beetles possess UV‐, blue‐, green‐, and red‐sensitive photoreceptors, and perceive color differently from humans. Jewel beetle photoreceptor signals were calculated for tree leaf and tree bark stimuli, representing feeding and oviposition sites of adult jewel beetles respectively. Artificial neural networks (ANNs) were trained to discriminate those stimuli using beetle photoreceptor signals, providing in silico models of the neural processing that might have evolved to drive behavior. ANNs using blue‐, green‐, and red‐sensitive photoreceptor inputs could classify these stimuli with very high accuracy (>99%). ANNs processed photoreceptor signals in an opponent fashion: increasing green‐sensitive photoreceptor signals promoted leaf classifications, while increasing blue‐ and red‐sensitive photoreceptor signals promoted bark classifications. Trained ANNs were fed photoreceptor signals calculated for traps, wherein they always classified green traps as leaves, but often classified purple traps as bark, indicating that these traps share salient features with different classes of tree stimuli from a beetle's eye view. A metric representing the photoreceptor opponent mechanism implicated by ANNs then explained catches of emerald ash borer, *Agrilus planipennis*, at differently colored traps from a previous field study. This analysis provides a hypothesized behavioral mechanism that can now guide the rational selection and improvement of jewel beetle traps.

## Graphical abstract

Jewel beetles can discriminate leaf feeding sites and bark oviposition sites based upon the opponent comparison of their blue, green, and red photoreceptor signals. Through this mechanism, green traps resemble leaves, and purple traps resemble bark, explaining their different attractiveness to males and females. A photoreceptor metric based on this mechanism predicts beetle catches at colored traps and provides a rational basis for trap selection or improvement.

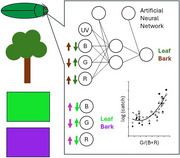

## Introduction

Jewel beetles (Coleoptera: Buprestidae) are colorful phytophagous insects with larvae that bore beneath tree bark, or occasionally within leaves or soft stems, and adults that feed on leaves or nectar. The family includes several significant, emerging, or potential pests of forest trees, including the emerald ash borer, *Agrilus planipennis*, which has devastated forestry since its accidental introduction to North America and Europe (Imrei *et al.*, 2020). Because populations of such pests establish quickly, efficient traps are essential to detect their presence (Imrei *et al.*, 2020), and Dodds *et al.* (2024) suggest that such traps can be designed by “thinking like a beetle” (i.e., by understanding beetle behavior and the sensory information guiding it, so that they can be exploited). However, doing so is a challenge because the differing sensory machinery and evolutionary pressures on jewel beetles mean that their perceptual worlds are very different from those of the humans seeking to understand them (von Uexküll, 2010; Caves *et al.*, 2019; Santer & Allen, 2024).

Several different trap designs currently exist for jewel beetle monitoring, including prism and multifunnel traps (Imrei *et al.*, 2020; Dodds *et al.*, 2024). These are generally green or purple to a human eye (or both in combination), and can be accompanied by odor lures (Imrei *et al.*, 2020; Dodds *et al.*, 2024). Across jewel beetle species there are differences in whether green or purple traps are more attractive, but for many species—including *A. planipennis*—green traps attract more beetles overall, while catches at purple traps are more biased toward females (Francese *et al.*, 2010; Imrei *et al.*, 2020). Understanding why jewel beetles are attracted to these trap colors, both functionally and mechanistically, could provide a rational basis for trap optimization (Santer & Allen, 2024).

In terrestrial habitats containing plants, natural reflectance spectra can be grouped into three types: (i) green leaves with a reflectance peak at approximately 555 nm (termed “leaf green”); (ii) most inorganic and many organic surfaces, including tree bark, dead vegetation, animal melanin pigments, rocks, and soil, where reflectance monotonically increases with wavelength (termed “gray‐red”); and (iii) signaling colors such as fruits, flowers, and ornaments that follow no common pattern but contrast against a leaf background (termed “leaf contrast”) (Osorio & Bossomaier, 1992). While jewel beetles also use color to identify mates (e.g., Wang *et al.*, 2022), for those species that feed on leaves as adults, leaf green stimuli would be characteristic of feeding and mating sites, while gray‐red stimuli would be characteristic of tree bark oviposition sites. Therefore, one possibility is that green traps resemble the former (hence attracting beetles of both sexes), and purple traps the latter (hence attracting ovipositing female beetles), within the perceptual world of a jewel beetle (c.f. Francese *et al.*, 2010).

**Fig. 1:**
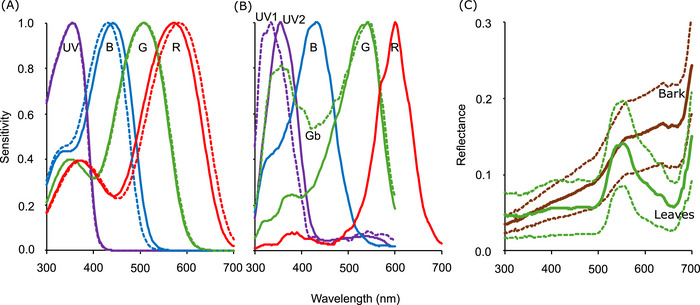
Spectra used to calculate jewel beetle photoreceptor signals. (A) Photoreceptor spectral sensitivity curves for *Chrysochroa mniszechii* (solid lines) and *Ch. rajah* (dotted lines) constructed using a template and adjustment for self‐screening (Goverdovskii *et al.*, 2000; Stavenga *et al.*, 2000), based upon *λ*max values measured electrophysiologically for opsins expressed in *Drosophila* (Sharkey *et al.*, 2023). Letters indicate photoreceptor types: UV = ultraviolet, B = blue, G = green, R = red. (B) Photoreceptor spectral sensitivity curves for *Coraebus undatus* based upon intracellular electrophysiological recordings (Meglič *et al.*, 2020). Figure is adapted with permission of the Company of Biologists Ltd from Meglič *et al.* (2020) (https://doi.org/10.1242/jeb.225920), and excluded from the CC license of the current work. Letters indicate photoreceptor types as above; Gb = broadband green. (C) Mean (solid lines) ± sample standard deviation (dotted lines) of the reflectance spectra of 80 oak leaves (green lines) and 80 oak bark samples (brown lines).

A beetle's photoreceptor signals provide the inputs from which its visual perceptions are formed, so in order to understand the attractiveness of different trap colors, they must be considered from this perspective (Santer & Allen, 2024). Ancestrally, insects possessed trichromatic visual systems with UV‐, short wavelength‐, and long wavelength‐sensitive opsins (Briscoe & Chittka, 2001), but beetles lost the short wavelength‐sensitive opsin mediating sensitivity to blue light (Sharkey *et al.*, 2017). Subsequent duplication of the retained opsins in jewel beetles has increased their complement of photoreceptor spectral types (Sharkey *et al.*, 2017; Sharkey *et al.*, 2023). By expression and characterization of the opsins of two *Chrysochroa* spp. in *Drosophila*, it was found that these species possess UV‐, blue‐, green‐, and orange‐sensitive photopigments (henceforth long‐wavelength‐sensitive photoreceptors will be referred to as “red‐sensitive” for simplicity; Fig. 1A) (Sharkey *et al.*, 2023). Intracellular electrophysiological recordings in *Coraebus undatus* revealed four corresponding spectral types of photoreceptor, but also identified an additional UV‐sensitive photoreceptor type, and a broadband type with sensitivity peaks in the UV and in the green (Fig. 1B) (Meglič *et al.*, 2020). These recordings suggested that photoreceptor sensitivity curves were shaped by screening resulting from the complex arrangement of rhabdomeres within an ommatidium (Gokan & Meyer‐Rochow, 1984; Meglič *et al.*, 2020).

One way to understand color attraction within the perceptual world of an insect is to build models relating calculated photoreceptor signals to behavioral responses: “photoreceptor‐based models” (Kelber, 2001; Kelber *et al.*, 2003). Artificial Neural Networks (ANNs) now provide an advanced way to build such models and understand the function and mechanism of insect behavior (Santer *et al.*, 2023). Herein, ANNs were trained to classify reflectance spectra from trees as either leaves or bark, based only upon calculated signals for jewel beetle photoreceptors. Because evolution also selects for neural mechanisms that efficiently serve behavioral demands, and because ANNs can be thought of as abstract mechanistic models of real nervous systems (Gurney, 2007), trained ANNs provide a working hypothesis regarding the sensory processing that might underlie jewel beetle behavior. After training, ANNs were challenged with photoreceptor signals for green and purple traps, in order to understand whether the different trap colors were misclassified as leaves or bark, and thus whether they shared salient features of the sensory information available to a jewel beetle. Finally, the value of the insights gained from ANNs in predicting the behavior of real jewel beetles was ascertained by using a simple photoreceptor metric to explain catches of *A. planipennis* from a previously published field study (Francese *et al.*, 2010).

## Materials and methods

### Reflectance spectra

Leaf and bark samples were collected from sessile oak (*Quercus petraea*) in woodland close to the Aberystwyth University campus (52°25′09.5″N, 4°04′12.8″W). Since pest jewel beetle species commonly prefer broadleaf trees, and a number of species are known to specialize on oaks (Imrei *et al.*, 2020), this species was chosen as a locally plentiful representative of a typical host tree. In total, 80 samples of living leaves, and 80 samples of bark, were collected to represent the range of coloration within those stimulus classes. The reflectance spectrum of each sample was measured using an Ocean Optics USB 4000 spectrometer, PX‐2 pulsed xenon light source flashing with a 30 ms period, bifurcated reflectance probe, and WS‐1‐SL standard (Ocean Optics Inc., Largo, FL, USA). The reflectance probe was angled at 45° to vertical to capture diffuse reflectance, and the distance between probe tip and sample was 6 mm. Integration time was 120 ms, boxcar width was 5, and 25 scans were averaged to smooth data. For each leaf, the reflectance of the adaxial and abaxial surface was measured and we noted variability in reflectance between leaf surfaces. To capture this variability while still maintaining a balanced sample of leaf and bark spectra, one measurement per leaf was randomly selected for analysis such that the final data set included 80 leaf reflectance spectra comprising 40 adaxial and 40 abaxial surfaces. Spectra were subsampled to achieve 2 nm wavelength resolution for the purposes of analysis, and are available in Data set S1.

**Fig. 2:**
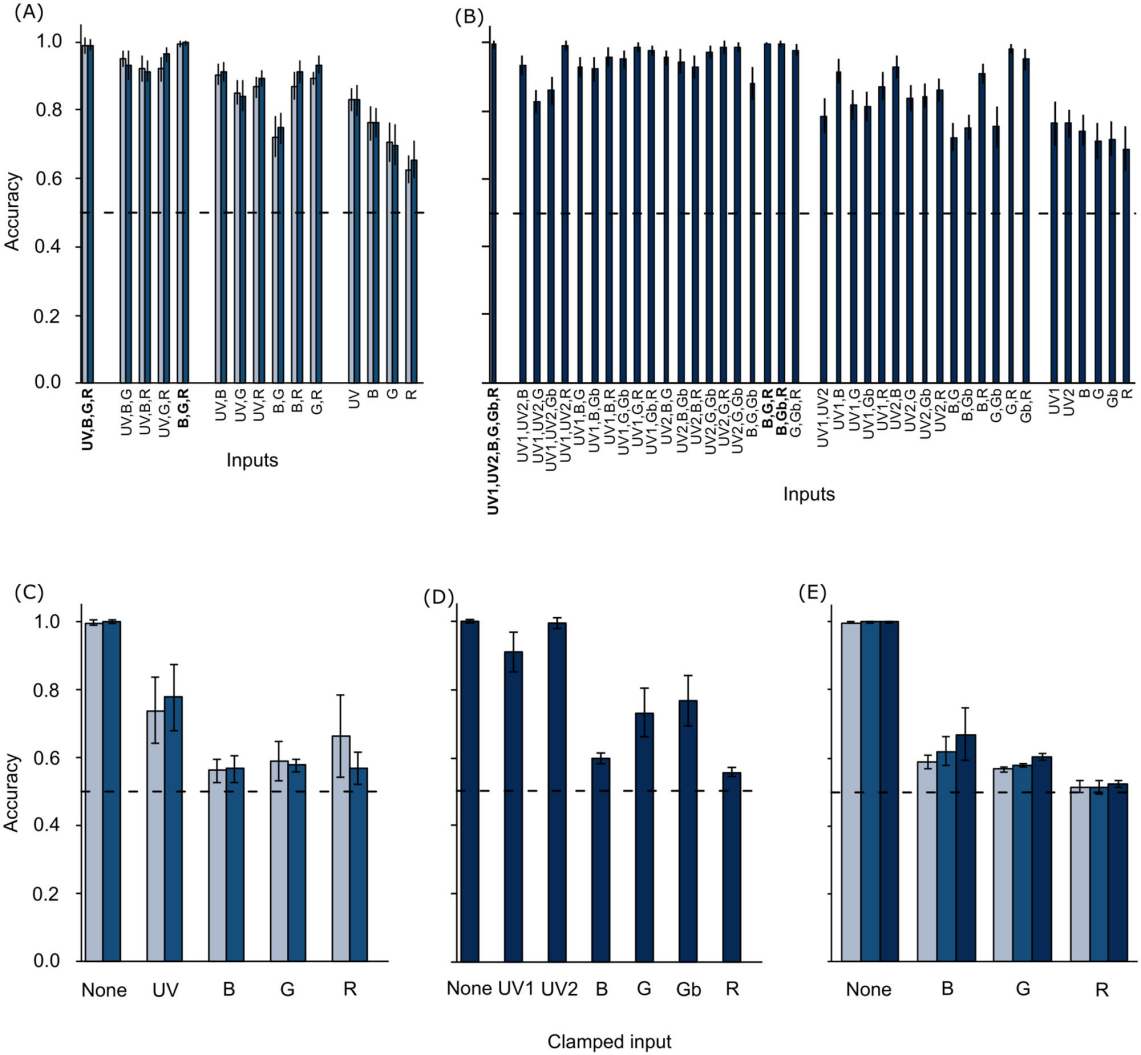
Evaluation of ANNs trained to distinguish “leaf” and “bark” stimuli. (A, B) The accuracy of trained ANNs in classifying a set of test stimuli not encountered during training, according to the number of photoreceptor excitation inputs they received. (A) ANNs using *Ch*. *mniszechii* (light blue bars) and *Ch. rajah* (medium blue bars) photoreceptor inputs. (B) ANNs using *C. undatus* photoreceptor inputs. (C−E) The accuracy of trained ANNs in classifying the complete data set of stimuli with the excitation value of individual photoreceptor inputs clamped to their median values. (C) ANNs receiving all four *Ch. mniszechii* (light blue bars) and *Ch. rajah* (medium blue bars) photoreceptor inputs. (D) ANNs receiving all six *C. undatus* photoreceptor inputs. (E) ANNs receiving only blue‐, green‐, and red‐sensitive photoreceptor inputs using *Ch*. *mniszechii* (light blue bars), *Ch. rajah* (medium blue bars), and *C. undatus* (dark blue bars) sensitivity curves. Each plot collates results for the best 20 ANNs of each type using means and sample standard deviations. Dashed lines indicate the level of classification success expected by chance. Letters indicate photoreceptor types as follows: UV = ultraviolet, B = blue, G = green, Gb = broadband green, R = red.

**Fig. 3:**
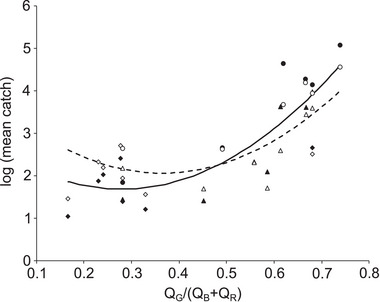
Relationship between jewel beetle catches recorded in a previous field study, and trap color represented using a metric based on the photoreceptor‐driven mechanism of stimulus classification implicated by ANNs. Data are mean catches of male (filled points) and female (open points) *A. planipennis* derived from Francese *et al.* (2010), with different symbols indicating the three experiments within that study. Q_G_/(Q_B_+Q_R_) is a metric representing the opponent comparison of photoreceptor responses implicated by ANNs, here calculated using *C. undatus* photoreceptor sensitivity functions. Higher values of this metric would be associated with leaf classifications by ANNs, and lower values associated with bark classifications by ANNs. All stimuli with a value > 0.4 are green to a human eye, and those with a value < 0.4 include purple, blue, and red traps. Quadratic relationships have been fitted for both males (intercept = 2.759 ± 1.153 (sem), *Z* = 2.392, *P* = 0.017; slope for metric = −7.698 ± 5.545, *Z* = −1.388, *P* = 0.165; slope for metric^2^ = 13.787 ± 5.890, *Z* = 2.341, *P* = 0.019; black line), and females (intercept = 3.909 ± 0.986, *Z* = 3.966, *P* < 0.001; slope for metric = −10.147 ± 4.613, *Z* = −2.200, *P* = 0.028; slope for metric^2^ = 13.899 ± 4.848, *Z* = 2.867, *P* = 0.004; dashed line).

In addition to tree spectra, spectra for the traps tested by Francese *et al.* (2010) were extracted from figures 1, 2, 3 of that publication at 2 nm wavelength resolution using DataThief software (Tummers, 2006). These included “Young Ash [YA] Green” and “Coroplast Purple” plastic traps, and “TSU Purple” painted traps. Color names are the reference names specified by Francese *et al.* (2010), wherein “TSU Purple” evokes earlier work on purple traps at Tennessee State University (Oliver *et al.*, 2002; Perkovich *et al.*, 2022; Perkovich *et al.*, 2023). Also included were a range of painted traps providing variations on purple and green spectra. “YA Green” had a leaf‐like green reflectance spectrum; “Coroplast” and “TSU” purples both had blue and red reflectance peaks, but while their blue peaks were similar, “TSU Purple” had a larger red peak (Francese *et al.*, 2010).

### Calculating photoreceptor signals

Photoreceptor signals were calculated for each characterized jewel beetle photoreceptor type in response to each reflectance spectrum. The quantum catch, *Q*, for each photoreceptor type was calculated by:

$$Q=\frac{\int_{300}^{700}I\left(\lambda \right)\cdot R_{s}\left(\lambda \right)\cdot S_{i}\left(\lambda \right)d\lambda }{\int_{300}^{700}I\left(\lambda \right)\cdot R_{b}\left(\lambda \right)\cdot S_{i}\left(\lambda \right)d\lambda },$$

where *I* is the irradiance spectrum on the stimulus and background, *R_s_* and *R_b_* are the reflectance spectra of the stimulus and predominant background, and *S* is the photoreceptor sensitivity spectrum for receptor type *i*. Leaf spectra provide the dominant background in most terrestrial habitats with living vegetation, so *R_b_* was the mean reflectance spectrum calculated across the sample of 80 leaves. *I* was the mean “open/cloudy” irradiance spectrum recorded by Endler (1993) and plotted in figure 1 of Santer *et al.* (2023), since emerald ash borer are more likely to colonize trees in open spaces and prefer to oviposit on sunlit bark (Timms *et al.*, 2006; Francese *et al.*, 2008). This “open/cloudy” spectrum is characteristic of open areas under sunny conditions, or any forest light environment under cloudy conditions (Endler, 1993). Analysis of equivalent data calculated using a woodland shade illuminant spectrum is presented in Tables S1−S4. The irradiance spectrum had units of photon flux, since energy units are irrelevant to studies of vision (Endler, 1990). We used three different sets of photoreceptor sensitivity spectra, *S_i_*, with the intention of identifying patterns that may apply broadly across jewel beetle species. Two sets of sensitivity spectra were calculated using a template (Goverdovskii *et al.*, 2000), *λ*max values determined for *Chrysochroa mniszechii* and *Ch. rajah* by expression of their opsins in *Drosophila* and subsequent electrophysiological characterization (Sharkey *et al.*, 2023), and adjustment for self‐screening (Stavenga *et al.*, 2000) (Fig. 1A). Jewel beetle photoreceptor sensitivity is shaped by possible coexpression of opsins or electrical coupling of photoreceptors, and by screening resulting from the complex arrangement of rhabdomeres within an ommatidium (Meglič *et al.*, 2020), which were not represented in these spectra. Thus, the third set of sensitivity spectra were extracted from electrophysiological recordings from *Coraebus undatus* (Meglič *et al.*, 2020), using DataThief software (Tummers, 2006) (Fig. 1B). All functions had 2 nm wavelength resolution.

Since photoreceptor responses relate nonlinearly to the number of quanta absorbed, values to represent photoreceptor excitation, *E*, were calculated to serve as input to ANNs:

$$E=\ln\left(Q\right).$$

### Training and evaluation of artificial neural networks

ANNs were created using the nnet package for R (Venables & Ripley, 2002), using the R script provided by Santer *et al.* (2023). All ANNs were fully connected, feedforward networks with an input layer, a hidden layer, and an output layer. For each ANN, the input layer comprised photoreceptor inputs calculated using sensitivity spectra for one of the jewel beetle species (*Ch. mniszechii*, *Ch. rajah*, or *C. undatus*). ANNs were trained using all four to six available photoreceptor inputs for a given species, and for subsets of those inputs to interrogate the importance of the different inputs to accurate classification. The hidden layer always had two artificial neurons, and the output layer had one neuron with logistic activation function, which produced a binary outcome with 0 corresponding to “bark,” and 1 corresponding to “leaf.”

Training of ANNs followed Santer *et al.* (2023). The stimulus data set was randomly split to use 60% of the stimuli for training and 40% for testing. 100 ANNs were trained on the training data using randomly determined starting weights, and maximum conditional likelihood to optimize performance. After training, maximum conditional likelihood was used to select the best model from the set of 100. This process was repeated 20 times using a unique random assortment of the stimuli into training and testing subsets. This procedure created an ensemble of 20 best‐fitting ANNs selected from 2000 that were trained in total. After training, the selected ANNs were tested using the retained test data (i.e., randomized subsampling cross‐validation), and the proportion of classifications that were correct (i.e., true positives plus true negatives) was used to quantify classification accuracy.

ANNs were tested to evaluate the mechanisms they used by the clamping method (Wang *et al.*, 2000), following Santer *et al.* (2023). First, each ANN classified the complete database of stimuli and its accuracy was recorded. The performance of the network was then reevaluated with one of its photoreceptor inputs clamped at the median value for that photoreceptor across all of the stimuli considered, and the other photoreceptor inputs presented to the network without alteration. If clamping substantially reduced an ANN's classification accuracy, then the clamped photoreceptor was an important part of the classification mechanism. The stimuli whose classifications changed as a result of the clamping procedure were then identified, and the difference between clamped and original excitation values for the photoreceptor in question calculated for each of these stimuli. The association between the sign of these differences and the likelihood of reclassification as a particular stimulus class was then investigated. This procedure was applied in turn to each photoreceptor type.

### Evaluating the appearance of green and purple traps

Beetle photoreceptor excitations for “YA Green,” “Coroplast Purple,” and “TSU Purple” *A. planipennis* (emerald ash borer) traps (as tested by Francese *et al.*, 2010) were fed to the 20 best‐fitting ANNs to understand how they might be perceived by a beetle. The percentage of models that made “leaf” versus “bark” classifications for a given trap was recorded, wherein 0% indicated that all 20 models made “bark” classifications, and 100% indicated that all 20 models made “leaf” classifications.

### Predicting catches of jewel beetles at colored traps

To evaluate whether insights from ANNs could be used to predict catches of real jewel beetles in field experiments, we used data from Francese *et al.* (2010). Francese *et al.* (2010) report catches of *A. planipennis* at sticky prism traps in a series of three experiments evaluating (a) various purple traps, (b) various green traps with maximum reflectance at differing wavelengths, and (c) various green traps with differing maximum reflectance. Catch data were provided in tables, from which we used the stated mean catch and adjusted it by the stated male:female ratio, to give separate mean catch values for males and females. Francese *et al.* (2010) used traps at ground level and mid‐canopy, but averaged catches across trap heights for two experiments. We averaged the mean catches at the two trap heights for the remaining experiment so that data were comparable. We log‐transformed mean catches for analysis.

ANNs suggested that leaves and bark could be classified based upon an opponent comparison of blue‐, green‐, and red‐sensitive photoreceptor signals (see Results). Therefore, for each of the stimulus reflectance spectra in Francese *et al.* (2010) we calculated a simple representation of this photoreceptor comparison as follows:

$$Q_{G}/\left(Q_{B}+Q_{R}\right),$$

where *Q_i_* is the quantum catch of the green, blue, and red photoreceptor respectively. Higher values of this metric indicate traps that cause relatively greater excitation in green‐ than blue‐ and red‐sensitive photoreceptors, and lower values indicate traps that cause relatively greater excitation in blue‐ and red‐ than green‐sensitive photoreceptors. We calculated this metric for each trap tested, using all three sets of jewel beetle photoreceptor sensitivity curves considered above.

We examined the relationship between the above photoreceptor metric and log‐transformed mean catches using generalized linear mixed models implemented in glmmTMB (Brooks *et al.*, 2017). These models specified a Gaussian distribution and identity link function, and a random intercept for experiment. We evaluated the relative fit of linear and quadratic relationships using AIC, and evaluated the overall fit of models using DHARMa (Hartig, 2022). We report data derived from *C. undatus* sensitivity functions in main text and provide the others in Table S5.

## Results

First, ANNs were trained to classify leaf versus bark stimuli (Fig. 1C) using jewel beetle photoreceptor signals. Regardless of which jewel beetle species’ photoreceptor sensitivity spectra were used, ANNs using all available inputs had very high accuracy in this task (> 98%; Fig. 2A, B). In such ANNs using four photoreceptor inputs based on *Chrysochroa* spp., clamping the signals of the blue‐, green‐, and red‐sensitive photoreceptors to their median values so that they provided no useful information greatly reduced classification accuracy, while clamping the signal of the UV‐sensitive photoreceptor had a lesser effect (Fig. 2C). For the ANN using six photoreceptor inputs based on those of *C. undatus*, clamping either UV‐sensitive photoreceptor signal had a negligible effect on classification accuracy, clamping either green‐sensitive photoreceptor caused an intermediate reduction in accuracy, and clamping the blue‐ or red‐sensitive photoreceptors greatly reduced classification accuracy (Fig. 2D). Thus, not all photoreceptor inputs were equally important to accurate classification in these ANNs and there appeared to be some redundancy.

While classification accuracy generally declined as ANNs received less photoreceptor inputs, ANNs receiving only blue‐, green‐, and red‐sensitive photoreceptor inputs achieved accuracy comparable to the ANNs using all available inputs, and exceeding that of ANNs with less inputs (>99%; Fig. 2A, B). In these models, clamping any photoreceptor signal to its median value greatly reduced classification accuracy toward the level expected by random chance, indicating that all were important to accurate classification (Fig. 2E). Thus, the signals of blue‐, green‐, and red‐sensitive photoreceptors were sufficient for highly accurate classification of tree stimuli.

Next, the mechanisms by which ANNs made these classifications were interrogated by examining the precise effect that clamping a given photoreceptor input had on classification. For ANNs using only blue‐, green‐, and red‐sensitive photoreceptor inputs, where the clamping operation increased the response of the green‐sensitive photoreceptor (and the broadband green‐sensitive photoreceptor of *C. undatus*) versus its original value for a given stimulus, 100% of the classifications that changed, changed from “bark” to “leaf.” Where clamping decreased the response of that photoreceptor, 100% of the classifications that changed, changed from “leaf” to “bark.” The opposite was true for the blue‐ or red‐sensitive photoreceptors: where clamping decreased their responses, the classifications that changed went exclusively from “bark” to “leaf”; but where clamping increased their responses, changes were from “leaf” to “bark.” The same patterns were evident for more complex ANNs using all available photoreceptor inputs, with the exception that for 3 out of 20 ANNs using the full set of *Ch. mniszechii* photoreceptor signals an increased red‐sensitive photoreceptor signal caused a mix of changes (2 ANNs), or changes solely from “bark” to “leaf” (1 ANN). Nevertheless, the predominant pattern was that ANNs processed photoreceptor signals in an opponent manner, with stronger green signals promoting leaf classifications, and stronger blue and red signals promoting bark classifications.

Trained ANNs were next challenged with photoreceptor signals calculated for purple and green traps used for *A. planipennis* monitoring (Table 1). All ANNs classified “YA Green” traps as “leaf,” and most classified “TSU Purple” as “bark.” However, there was variation in the way that “Coroplast Purple” was classified both between and within the different types of ANN. This result suggests that from a beetle's point of view, “TSU purple” traps share salient features with bark, and “YA Green” traps share salient features with leaves.

**Table 1:** The percentage of trained ANNs that classified jewel beetle traps as “leaf” rather than “bark.” Data are the percentage of “leaf” classifications across the 20 best ANNs of each type, for full models using all photoreceptor inputs, and in brackets reduced models using only blue‐, green‐, and red‐sensitive photoreceptor inputs. 100% indicates that all ANNs classified a stimulus as “leaf”; 0% indicates that all ANNs classified a stimulus as “bark.” Color names are those used by Francese *et al.* (2010)

Inputs	“TSU Purple”	“Coroplast Purple”	“YA Green”
*Ch*. *mniszechii*	0% (30%)	0% (90%)	100% (100%)
*Ch. rajah*	5% (5%)	5% (70%)	100% (100%)
*C. undatus*	5% (0%)	20% (30%)	100% (100%)

Finally, to evaluate whether insights from ANNs could aid in trap optimization, we analyzed preexisting data on catches of *A. planipennis* at a variety of green and purple traps (Francese *et al.*, 2010). For each of the previously tested traps we calculated a simple metric to represent the opponent comparison of green‐ versus blue‐ and red‐sensitive photoreceptors suggested by ANNs [*Q_G_*/(*Q_B_* +*Q_R_*)], for which higher values indicate traps causing relatively greater excitation in green‐ than blue‐ and red‐sensitive photoreceptors (resembling stimuli that would cause ANNs to make leaf classifications), and lower values indicate traps causing relatively greater excitation in blue‐ and red‐ than green‐sensitive photoreceptors (resembling stimuli that would cause ANNs to make bark classifications). Log mean catches of males and females had a quadratic relationship with this metric calculated using *C. undatus* photoreceptor sensitivity spectra (Fig. 3). For females, catches increased as traps provided stronger photoreceptor signals associated with leaf classification, and to a lesser extent also with stronger photoreceptor signals associated with bark classification (Fig. 3, dashed line). For males, the increase in catches with photoreceptor signals associated with bark classification was less marked (Fig. 3, black line), and when the metric was calculated using photoreceptor sensitivity functions for other species, log mean catches of males were adequately fitted by a linear relationship (Table S5).

## Discussion

In this work, ANNs were trained to classify tree leaf and tree bark stimuli based on jewel beetle photoreceptor signals, revealing the optimal way in which those signals could be processed to classify behaviorally relevant stimuli, and providing a hypothesis for the neural mechanism that might drive jewel beetle behavior in that context. ANNs with blue‐, green, and red‐sensitive photoreceptor inputs could classify stimuli with high accuracy, suggesting that the diversified photoreceptor machinery of jewel beetles could provide a functional advantage in this context. Trained ANNs distinguished leaves and bark by processing those photoreceptor inputs in an opponent fashion, and as a result classified green traps as leaves, and tended to classify some purple traps as bark. This suggests that these traps share salient features of different natural stimuli through the eyes of a beetle, explaining their differing attractiveness to males and females. A photoreceptor metric representing the principle by which ANNs made leaf and bark classifications could explain catches of real jewel beetles at differently colored traps obtained in a previous field study (Francese *et al.*, 2010), providing evidence for the plausibility of the hypothesized behavioral mechanism, and demonstrating how such a mechanistic understanding of beetle behavior can provide a rational basis for trap improvement.

This study modeled the perception of tree stimuli by jewel beetles in a generic rather than species‐specific way. Jewel beetles possess at least four opsins, including one with long wavelength sensitivity which is relatively unusual among insects (Sharkey *et al.*, 2017; van der Kooi *et al.*, 2021; Sharkey *et al.*, 2023). However, electrophysiological recordings for *C. undatus* suggest that photoreceptor sensitivity is further shaped by possible coexpression of opsins or electrical coupling of photoreceptors, and by screening resulting from the complex arrangement of rhabdomeres within an ommatidium (Gokan & Meyer‐Rochow, 1984; Meglič *et al.*, 2020). While we used photoreceptor sensitivity curves for *C. undatus* that incorporated such effects (Meglič *et al.*, 2020), we also used sensitivity curves for *Ch. mniszechii* and *Ch. rajah* based only on photopigment absorbance ascertained by expressing the relevant opsins in *Drosophila* (Sharkey *et al.*, 2023). Nevertheless, results were comparable across all three sets of photoreceptor data, demonstrating that our overall conclusions are valid for a generic jewel beetle visual system, and robust to species differences in spectral sensitivity. In addition, our analyses considered discrimination of leaf and bark stimuli in a single tree species, while jewel beetle species are known to vary in host preferences (Imrei *et al.*, 2020). Our approach was valid since the reflectance spectra of leaves and bark of different plant species conform to well‐described patterns (Osorio & Bossomaier, 1992), but it remains to be investigated how a jewel beetle of a given species might discriminate its preferred host tree species (e.g., Campbell & Borden, 2005).

ANNs receiving blue‐, green‐, and red‐sensitive photoreceptor signals as input were highly accurate in distinguishing leaf and bark stimuli, with > 99% accuracy. Such ANNs attained considerably greater accuracy than those using only UV‐ and green‐sensitive photoreceptor inputs typical of ancestral beetles (ca. 80%−85% accuracy), or those using only the green‐sensitive photoreceptor input (ca. 70% accuracy). This supports the hypothesis that more effective separation of leaf and bark stimuli may have been a selective pressure that favored the duplication of opsins in jewel beetles (Sharkey *et al.*, 2017; Sharkey *et al.*, 2023). In *A. planipennis*, electroretinograms suggested greater red sensitivity in females than males (Crook *et al.*, 2009), and since females must identify tree bark for oviposition, this may suggest an adaptation of the female eye in presence or abundance of these photoreceptors. However, no such sexual dimorphism was found in the photoreceptors of *C. undatus* through intracellular recordings (Meglič *et al.*, 2020). In addition to identifying leaf and bark stimuli, possession of a long wavelength‐sensitive photoreceptor has been shown to enhance the chromatic contrast of conspecific jewel beetles against vegetation backgrounds, suggesting that there are likely to be several advantages to long wavelength sensitivity (Wang *et al.*, 2022).

Clamping individual photoreceptor inputs to trained ANNs revealed that blue‐, green‐, and red‐sensitive photoreceptor inputs were critical to accurate classification, though the two types of green‐sensitive photoreceptor of *C. undatus* appeared to be redundant when both were provided as input to the same ANN. ANNs processed these photoreceptor inputs in an opponent fashion, with increasing green‐sensitive photoreceptor responses promoting leaf classifications, and increasing blue‐ and red‐sensitive photoreceptor responses promoting bark classifications. This organization resembles the situation in swallowtail butterflies, *Papilio aegeus*, where possession of a red‐sensitive photoreceptor allows butterflies to discriminate green stimuli indicative of young leaves (Kelber, 1999). It also accords with field experimentation on *A. planipennis* and *Chrysobothris femorata* wherein both the blue and red reflectance peaks of purple traps were important determinants of their attractiveness (Francese *et al.*, 2010; Perkovich *et al.*, 2022).

By training ANNs, the optimal way to relate photoreceptor signals to stimulus classification was identified. Selection on real nervous systems should also favor efficient neural processing serving behavior, and ANNs can be seen as modeling real nervous systems in an abstract mechanistic way (Gurney, 2007), so the solutions found by ANNs provide a working hypothesis as to the mechanism that might underlie jewel beetle behavior (c.f. Santer *et al.*, 2023). Trained ANNs always classified “YA Green” traps as leaves, but results for the two purple traps considered were inconsistent. “TSU Purple” traps were most often classified as bark, while there was variation between and within ANN types in the classification of “Coroplast Purple” traps. This is likely to be because “Coroplast Purple” has a lesser red reflectance peak than “TSU purple,” and was found to be less attractive than it in a previous field study on *A. planipennis* (Francese *et al.*, 2010). Nevertheless, these results suggest that green and purple traps share salient features with different classes of tree stimuli, and given that females oviposit on bark, this helps explain the female bias of *A. planipennis* catches at purple traps (Francese *et al.*, 2010). Aligning with this, previous experiments on *Chrysobothris femorata* found that a combination of tall, thin traps resembling tree sapling trunks, and purple coloration incorporating both blue and red reflectance peaks, attracted larger numbers of beetles (Oliver *et al.*, 2002; Perkovich *et al.*, 2022; Perkovich *et al.*, 2023). This understanding provides a rational basis for the combination of olfactory and visual cues in trap design. A variety of olfactory lures including green leaf, bark oil, and pheromone lures have proved effective in jewel beetle traps (Imrei *et al.*, 2020). An improved understanding of how colored traps might be perceived by beetles permits olfactory lures to be chosen to enhance the illusion that a trap is an attractive leaf or section of bark, and thus improve trap effectiveness.

Recently, photoreceptor‐based models have been used to explain catches of pest insects at colored targets, and those models have provided a rational way to optimize targets for improved performance (Santer, 2014; Santer, 2017; Santer *et al.*, 2019; Santer *et al.*, 2021; Dearden *et al.*, 2023; Santer & Allen, 2024). Because insect traps often work by resembling or presenting super‐normal versions of behaviorally relevant natural stimuli, hypotheses about the photoreceptor mechanisms that classify those natural stimuli can reveal the sensory features likely to be important in guiding behavior, and thus can also provide insights useful for trap optimization. Here, catches of *A. planipennis* at variously colored traps could be explained by a photoreceptor metric representing the opponent mechanism of stimulus classification suggested by ANNs. For females, catches increased with increasingly more leaf‐like, and to a lesser extent, increasingly more bark‐like traps. For males, increased catches at bark‐like traps were less evident, presumably reflecting a requirement for both sexes to feed and mate in the tree canopy, and for females to oviposit on bark. Such metrics can guide trap improvement by providing a rational basis to select between available trap colors (c.f. Dearden *et al.*, 2023), and a principle to guide the deliberate creation of more attractive colors (c.f. Santer, 2017; Santer *et al.*, 2019). Based on these findings, a shade of green that maximized excitation of green‐sensitive photoreceptors, but minimized excitation of blue‐ and red‐sensitive photoreceptors, should provide a more effective trap for male and female jewel beetles. Conversely, a color that maximized excitation of blue‐ and red‐sensitive photoreceptors and minimized excitation of the green‐sensitive photoreceptor might better attract ovipositing females.

## Disclosure

RS currently supervises a PhD student funded by Forest Research, UK. Otherwise, the authors declare no conflict of interests.

## Supporting information




**Table S1** Mean ± sample standard deviation classification accuracy with inputs clamped to their median value for ANNs receiving different types and number of photoreceptor inputs, calculated using *Ch. mniszechii* or *Ch. rajah* spectral sensitivity functions, and different illuminant spectra. Test data comprise the entire data set.
**Table S2** Mean ± sample standard deviation classification accuracy with inputs clamped to their median value for ANNs receiving different types and numbers of photoreceptor inputs, calculated using *C. undatus* spectral sensitivity functions, and different illuminant spectra. Test data comprise the entire data set.
**Table S3** The effect of clamping different photoreceptor inputs on classification by ANNs, according to whether clamping increased or decreased that photoreceptor signal for a given stimulus (Inc. = increased, Dec. = decreased).
**Table S4** The percentage of ANNs that classified a given trap as “leaf.”
**Table S5** Relationships between *A. planipennis* catches recorded by Francese *et al.* (2010), and the Q_G_/(Q_B_+Q_R_) metric calculated using sensitivity functions for *Ch. mniszechii*, *Ch. rajah*, and *C. undatus*.

**Data set S1** Reflectance spectra for oak leaves and bark used to calculate jewel beetle photoreceptor responses, and calculated photoreceptor responses used as input to ANNs.

## References

[ins13496-bib-0001] Briscoe, A.D. and Chittka, L. (2001) The evolution of color vision in insects. Annual Review of Entomology, 46, 471–510. 10.1146/annurev.ento.46.1.471 11112177

[ins13496-bib-0002] Brooks, M.E. , Kristensen, K. , van Benthem, K.J. , Magnusson, A. , Berg, C.W. , Nielsen, A. *et al.* (2017) glmmTMB balances speed and flexibility among packages for zero‐inflated generalized linear mixed modeling. R Journal, 9, 378–400.

[ins13496-bib-0003] Campbell, S.A. and Borden, J.H. (2005) Bark reflectance spectra of conifers and angiosperms: implications for host discrimination by coniferophagous bark and timber beetles. Canadian Entomologist, 137, 719–722.

[ins13496-bib-0004] Caves, E.M. , Nowicki, S. and Johnsen, S. (2019) Von Uexküll revisited: addressing human biases in the study of animal perception. Integrative and Comparative Biology, 59, 1451–1462. 31127268 10.1093/icb/icz073

[ins13496-bib-0005] Crook, D.J. , Francese, J.A. , Zylstra, K.E. , Fraser, I. , Sawyer, A.J. , Bartels, D.W. *et al*. (2009) Laboratory and field response of the emerald ash borer (Coleoptera: Buprestidae), to selected regions of the electromagnetic spectrum. Journal of Economic Entomology, 102, 2160–2169. 20069845 10.1603/029.102.0620

[ins13496-bib-0006] Dearden, A.E. , Wood, M.J. , Frend, H.O. , Butt, T.M. and Allen, W.L. (2023) Visual modelling can optimise the appearance and capture efficiency of sticky traps used to manage insect pests. Journal of Pest Science, 97, 469–479.

[ins13496-bib-0007] Dodds, K.J. , Sweeney, J. , Francese, J.A. , Besana, L. and Rassati, D. (2024) Factors affecting catches of bark beetles and woodboring beetles in traps. Journal of Pest Science, 97, 1767–1793.

[ins13496-bib-0008] Endler, J.A. (1990) On the measurement and classification of colour in studies of animal colour patterns. Biological Journal of the Linnean Society, 41, 315–352.

[ins13496-bib-0009] Endler, J.A. (1993) The color of light in forests and its implications. Ecological Monographs, 63, 1–27.

[ins13496-bib-0010] Francese, J.A. , Crook, D.J. , Fraser, I. , Lance, D.R. , Sawyer, A.J. and Mastro, V.C. (2010) Optimization of trap color for emerald ash borer (Coleoptera: Buprestidae). Journal of Economic Entomology, 103, 1235–1241. 20857732 10.1603/ec10088

[ins13496-bib-0011] Francese, J.A. , Oliver, J.B. , Fraser, I. , Lance, D.R. , Youssef, N. , Sawyer, A.J. *et al.* (2008) Influence of trap placement and design on capture of the emerald ash borer (Coleoptera: Buprestidae). Journal of Economic Entomology, 101, 1831–1837. 19133464 10.1603/0022-0493-101.6.1831

[ins13496-bib-0012] Gokan, N. and Meyer‐Rochow, B. (1984) Fine‐structure of the compound eye of the buprestid beetle *Curis caloptera* (Coleoptera, Buprestidae). Zeitschrift für mikroskopisch‐anatomische Forschung, Leipzig, 98, 17–35. 6720018

[ins13496-bib-0013] Goverdovskii, V.I. , Fyhrquist, N. , Reuter, T. , Kuzmin, D.G. and Donner, K. (2000) In search of the visual pigment template. Visual Neuroscience, 17, 509–528. 11016572 10.1017/s0952523800174036

[ins13496-bib-0014] Gurney, K. (2007) Neural networks for perceptual processing: from simulation tools to theories. Philosophical Transactions of the Royal Society B, 362, 339–353. 10.1098/rstb.2006.1962 PMC2323553 17255023

[ins13496-bib-0015] Hartig, F. (2022) DHARMa: Residual diagnostics for hierarchical (multi‐level/mixed) regression models. R package version 0.4.6. http://florianhartig.github.io/DHARMa/.

[ins13496-bib-0016] Imrei, Z. , Lohonyai, Z. , Csóka, G. , Muskovits, J. , Szanyi, S. , Vétek, G. *et al.* (2020) Improving trapping methods for buprestid beetles to enhance monitoring of native and invasive species. Forestry, 93, 254–264.

[ins13496-bib-0017] Kelber, A. (1999) Ovipositing butterflies use a red receptor to see green. Journal of Experimental Biology, 202, 2619–2630. 10482721 10.1242/jeb.202.19.2619

[ins13496-bib-0018] Kelber, A. (2001) Receptor based models for spontaneous colour choices in flies and butterflies. Entomologia Experimentalis et Applicata, 99, 231–244.

[ins13496-bib-0019] Kelber, A. , Vorobyev, M. and Osorio, D. (2003) Animal colour vision—behavioural tests and physiological concepts. Biological Reviews, 78, 81–118. 12620062 10.1017/s1464793102005985

[ins13496-bib-0020] Meglič, A. , Ilić, M. , Quero, C. , Arikawa, K. and Belušič, G. (2020) Two chiral types of randomly rotated ommatidia are distributed across the retina of the flathead oak borer *Coraebus undatus* (Coleoptera: Buprestidae). Journal of Experimental Biology, 223, jeb225920. 32532862 10.1242/jeb.225920

[ins13496-bib-0021] Oliver, J.B. , Youssef, N. , Fare, D. , Halcomb, M. , Scholl, S. Klingeman, W. *et al.* (2002) Monitoring buprestid borers in production nursery areas. In Proceedings of the 29th Annual Meeting of the Tennessee Entomological Society (ed. G. Haun ), pp. 17–23. Nashville, TN.

[ins13496-bib-0022] Osorio, D. and Bossomaier, T.R.J. (1992) Human cone‐pigment spectral sensitivities and the reflectances of natural surfaces. Biological Cybernetics, 67, 217–222. 1498187 10.1007/BF00204394

[ins13496-bib-0023] Perkovich, C.L. , Addesso, K.M. , Basham, J.P. , Fare, D.C. , Youssef, N.N. and Oliver, J.B. (2022) Effects of color attributes on trap capture rates of *Chrysobothris femorata* (Coleoptera: Buprestidae) and related species. Environmental Entomology, 51, 737–746. 35762287 10.1093/ee/nvac038 PMC9389425

[ins13496-bib-0024] Perkovich, C.L. , Oliver, J.B. , Addesso, K.M. , Basham, J.P. and Youssef, N.N. (2023) Effects of trap shape, size, and color variations on capture rates of *Chrysobothris* (Coleoptera: Buprestidae) and related buprestids. Florida Entomologist, 106, 63–66.

[ins13496-bib-0025] Santer, R.D. (2014) A colour opponent model that explains tsetse fly attraction to visual baits and can be used to investigate more efficacious bait materials. PLoS Neglected Tropical Diseases, 8, e3360. 25473844 10.1371/journal.pntd.0003360 PMC4256293

[ins13496-bib-0026] Santer, R.D. (2017) Developing photoreceptor‐based models of visual attraction in riverine tsetse, for use in the engineering of more‐attractive polyester fabrics for control devices. PLoS Neglected Tropical Diseases, 11, e0005448. 28306721 10.1371/journal.pntd.0005448 PMC5371378

[ins13496-bib-0027] Santer, R.D. , Akanyeti, O. , Endler, J.A. , Galván, I. and Okal, M.N. (2023) Why are biting flies attracted to blue objects? Proceedings of the Royal Society B, 290, 20230463. 37357856 10.1098/rspb.2023.0463 PMC10291711

[ins13496-bib-0028] Santer, R.D. and Allen, W.L. (2024) Optimising the colour of traps requires an insect's eye view. Pest Management Science, 80, 931–934. 37755337 10.1002/ps.7790

[ins13496-bib-0029] Santer, R.D. , Okal, M.N. , Esterhuizen, J. and Torr, S.J. (2021) Evaluation of improved coloured targets to control riverine tsetse in East Africa: a Bayesian approach. PLoS Neglected Tropical Diseases, 15, e0009463. 34153040 10.1371/journal.pntd.0009463 PMC8216509

[ins13496-bib-0030] Santer, R.D. , Vale, G.A. , Tsikire, D. and Torr, S.J. (2019) Optimising targets for tsetse control: taking a fly's‐eye‐view to improve the colour of synthetic fabrics. PLoS Neglected Tropical Diseases, 13, e0007905. 31830039 10.1371/journal.pntd.0007905 PMC6907749

[ins13496-bib-0031] Sharkey, C.R. , Blanco, J. , Lord, N.P. and Wardill, T.J. (2023) Jewel beetle opsin duplication and divergence is the mechanism for diverse spectral sensitivities. Molecular Biology and Evolution, 40, msad023. 36721951 10.1093/molbev/msad023 PMC9937044

[ins13496-bib-0032] Sharkey, C.R. , Fujimoto, M.S. , Lord, N.P. , Shin, S. , McKenna, D.D. , Suvorov, A. *et al.* (2017) Overcoming the loss of blue sensitivity through opsin duplication in the largest animal group, beetles. Scientific Reports, 7, 8. 28127058 10.1038/s41598-017-00061-7 PMC5428366

[ins13496-bib-0033] Stavenga, D.G. , Oberwinkler, J. and Postma, M. (2000) Modeling primary visual processes in insect photoreceptors. In Handbook of Biological Physics, Volume 3. Molecular Mechanisms in Visual Transduction (eds. D.G. Stavenga , W.J. DeGrip & E.N. Pugh Jr ), pp. 527–574. Elsevier, Amsterdam.

[ins13496-bib-0034] Timms, L.L. , Smith, S.M. and De Groot, P. (2006) Patterns in the within‐tree distribution of the emerald ash borer *Agrilus planipennis* (Fairmaire) in young, green‐ash plantations of south‐western Ontario, Canada. Agricultural and Forest Entomology, 8, 313–321.

[ins13496-bib-0035] Tummers, B. (2006) Data Thief III. https://datathief.org/.

[ins13496-bib-0036] van der Kooi, C.J. , Stavenga, D.G. , Arikawa, K. , Belušič, G. and Kelber, A. (2021) Evolution of insect color vision: From spectral sensitivity to visual ecology. Annual Review of Entomology, 66, 435–461. 10.1146/annurev-ento-061720-071644 32966103

[ins13496-bib-0037] Venables, W.N. and Ripley, B.D. (2002) Modern Applied Statistics with S, Fourth edition, Springer, New York.

[ins13496-bib-0038] von Uexküll, J. (2010) A Foray into the Worlds of Animals and Humans with a Theory of Meaning ( J.D. O'Neil , Trans.). University of Minnesota Press, Minneapolis & London (original work published 1934 and 1940).

[ins13496-bib-0039] Wang, L.Y. , Stuart‐Fox, D. , Walker, G. , Roberts, N.W. and Franklin, A.M. (2022) Insect visual sensitivity to long wavelengths enhances colour contrast of insects against vegetation. Scientific Reports, 12, 982. 35046431 10.1038/s41598-021-04702-w PMC8770459

[ins13496-bib-0040] Wang, W. , Jones, P. and Partridge, D. (2000) Assessing the impact of input features in a feedforward neural network. Neural Computing and Applications, 9, 101–112.

