# Supplementary material for: Using artificial neural networks to explain the attraction of jewel beetles (Coleoptera: Buprestidae) to colored traps
Source: Insect Sci. 2025 Jan 16;33(1):325–35. doi: 10.1111/1744-7917.13496 (PMC12905469; doi:10.1111/1744-7917.13496)
Supplement: Supplementary file 1 — Table S1 Mean ± sample standard deviation classification accuracy with inputs clamped to their median value for ANNs receiving different types and number of photoreceptor inputs, calculated using Ch. mniszechii or Ch. rajah spectral sensitivity functions, and different illuminant spectra. Test data comprise the entire data set. Table S2 Mean ± sample standard deviation classification accuracy with inputs clamped to their median value for ANNs receiving different types and numbers of photoreceptor inputs, calculated using C. undatus spectral sensitivity functions, and different illuminant spectra. Test data comprise the entire data set. Table S3 The effect of clamping different photoreceptor inputs on classification by ANNs, according to whether clamping increased or decreased that photoreceptor signal for a given stimulus (Inc. = increased, Dec. = decreased). Table S4 The percentage of ANNs that classified a given trap as “leaf.” Table S5 Relationships between A. planipennis catches recorded by Francese et al. (2010), and the QG/(QB+QR) metric calculated using sensitivity functions for Ch. mniszechii, Ch. rajah, and C. undatus. [file INS-33-325-s001.docx]

**Supplementary results**

Results presented in the accompanying manuscript are based on photoreceptor signals calculated using an open/cloudy illuminant spectrum (Endler, 1993). Repeating the work using photoreceptor signals calculated using the woodland shade illuminant spectrum originally recorded by Endler (1993) and presented in Santer et al. (2023), yielded similar results (tables S1-S4).

In the accompanying manuscript, a photoreceptor metric calculated using *C. undatus* spectral sensitivity curves was used to explain emerald ash borer catches in a previous field study (Francese et al., 2010). Here, equivalent analyses using spectral sensitivity functions for other buprestid species are reported (table S5).

**Table S1.** Mean ± sample standard deviation classification accuracy with inputs clamped to their median value for ANNs receiving different types and number of photoreceptor inputs, calculated using different illuminant spectra. Test data comprise the entire dataset.

| Inputs | Illuminant | No clamping | UV clamped | B clamped | G clamped | R clamped |
| --- | --- | --- | --- | --- | --- | --- |
| *Ch. mniszechii*; UV, B, G, R | Open/Cloudy | 0.995±0.010 | 0.738±0.099 | 0.560±0.033 | 0.589±0.057 | 0.663±0.119 |
| *Ch. mniszechii*; B, G, R | Open/Cloudy | 0.998±0.004 | | 0.587±0.020 | 0.566±0.008 | 0.515±0.018 |
| *Ch. mniszechii*; UV, B, G, R | Woodland | 0.993±0.009 | 0.677±0.024 | 0.526±0.016 | 0.574±0.018 | 0.658±0.109 |
| *Ch. mniszechii*; B, G, R | Woodland | 0.997±0.007 | | 0.554±0.015 | 0.542±0.008 | 0.515±0.023 |
| *Ch. rajah*; UV, B, G, R | Open/Cloudy | 0.997±0.006 | 0.775±0.095 | 0.566±0.039 | 0.577±0.018 | 0.570±0.047 |
| *Ch. rajah*; B, G, R | Open/Cloudy | 0.999±0.003 | | 0.619±0.042 | 0.577±0.005 | 0.514±0.020 |
| *Ch. rajah*; UV, B, G, R | Woodland | 0.996±0.006 | 0.718±0.086 | 0.559±0.036 | 0.573±0.017 | 0.605±0.076 |
| *Ch. rajah*; B, G, R | Woodland | 0.997±0.006 | | 0.604±0.039 | 0.569±0.016 | 0.520±0.014 |

**Table S2.** Mean ± sample standard deviation classification accuracy with inputs clamped to their median value for ANNs receiving different types and numbers of photoreceptor inputs, calculated using different illuminant spectra. Test data comprise the entire dataset.

| Inputs | Illuminant | No clamping | UV1 clamped | UV1 clamped | B clamped | G clamped | Gb clamped | R clamped |
| --- | --- | --- | --- | --- | --- | --- | --- | --- |
| *C. undatus*;  UV1, UV2, B, G, Gb, R | Open/Cloudy | 0.998±0.005 | 0.911±0.058 | 0.995±0.015 | 0.598±0.016 | 0.732±0.069 | 0.768±0.073 | 0.556±0.013 |
| *C. undatus*; B, G, R | Open/Cloudy | 1.000±0.001 | |  | 0.669±0.077 | 0.603±0.009 | | 0.524±0.011 |
| *C. undatus*; B, Gb, R | Open/Cloudy | 0.999±0.004 | |  | 0.622±0.065 | | 0.606±0.007 | 0.538±0.013 |
| *C. undatus*;  UV1, UV2, B, G, Gb, R | Woodland | 0.997±0.006 | 0.846±0.079 | 0.988±0.024 | 0.590±0.047 | 0.691±0.065 | 0.733±0.069 | 0.589±0.068 |
| *C. undatus*; B, G, R | Woodland | 1.000±0.001 | |  | 0.639±0.075 | 0.593±0.010 | | 0.520±0.010 |
| *C. undatus*; B, Gb, R | Woodland | 0.997±0.007 | |  | 0.600±0.039 |  | 0.588±0.010 | 0.536±0.012 |

**Table S3.** The effect of clamping different photoreceptor inputs on classification by ANNs, according to whether clamping increased or decreased that photoreceptor signal for a given stimulus (Inc. = increased, Dec. = decreased). Data are mean ± sample standard deviation proportions of changed classifications that changed from ‘bark’ to ‘leaf’. A value of 1 indicates that all classifications that changed, changed from ‘bark’ to ‘leaf’. A value of 0 indicates that all classifications that changed, changed from ‘leaf’ to ‘bark’.

| Inputs | Illuminant | Inc. B | Inc. G | Inc. Gb | Inc. R | Dec. B | Dec. G | Dec. Gb | Dec. R |
| --- | --- | --- | --- | --- | --- | --- | --- | --- | --- |
| *Ch. mniszechii*; UV, B, G, R | Open/Cloudy | 0±0 | 1±0 |  | 0.095±0.257 | 1±0 | 0±0 |  | 1±0 |
| *Ch. mniszechii*; B, G, R | Open/Cloudy | 0±0 | 1±0 |  | 0±0 | 1±0 | 0±0 |  | 1±0 |
| *Ch. mniszechii*; UV, B, G, R | Woodland | 0±0 | 1±0 |  | 0.091±0.281 | 1±0 | 0±0 |  | 0.998±0.010 |
| *Ch. mniszechii*; B, G, R | Woodland | 0±0 | 1±0 |  | 0±0 | 1±0 | 0±0 |  | 1±0 |
| *Ch. rajah*; UV, B, G, R | Open/Cloudy | 0±0 | 1±0 |  | 0±0 | 1±0 | 0±0 |  | 1±0 |
| *Ch. rajah*; B, G, R | Open/Cloudy | 0±0 | 1±0 |  | 0±0 | 1±0 | 0±0 |  | 1±0 |
| *Ch. rajah*; UV, B, G, R | Woodland | 0±0 | 1±0 |  | 0±0 | 1±0 | 0±0 |  | 1±0 |
| *Ch. rajah*; B, G, R | Woodland | 0.039±0.174 | 1±0 |  | 0±0 | 1±0 | 0±0 |  | 1±0 |
| *C. undatus*; UV1, UV2, B, G, Gb, R | Open/Cloudy | 0±0 | 1±0 | 1±0 | 0±0 | 1±0 | 0±0 | 0±0 | 1±0 |
| *C. undatus*; B, G, R | Open/Cloudy | 0±0 | 1±0 |  | 0±0 | 1±0 | 0±0 |  | 1±0 |
| *C. undatus*; B, Gb, R | Open/Cloudy | 0±0 |  | 1±0 | 0±0 | 1±0 |  | 0±0 | 1±0 |
| *C. undatus*; UV1, UV2, B, G, Gb, R | Woodland | 0±0 | 1±0 | 1±0 | 0.025±0.112 | 1±0 | 0±0 | 0±0 | 1±0 |
| *C. undatus*; B, G, R | Woodland | 0±0 | 1±0 |  | 0±0 | 1±0 | 0±0 |  | 1±0 |
| *C. undatus*; B, Gb, R | Woodland | 0±0 |  | 1±0 | 0±0 | 1±0 |  | 0±0 | 1±0 |

**Table S4.** The percentage of ANNs that classified a given trap as ‘leaf’.

| Inputs | Illuminant | ‘TSU Purple’ | ‘Coroplast Purple’ | ‘YA Green’ |
| --- | --- | --- | --- | --- |
| *Ch. mniszechii*; UV, B, G, R | Open/Cloudy | 0% | 0% | 100% |
| *Ch. mniszechii*; B, G, R | Open/Cloudy | 30% | 90% | 100% |
| *Ch. mniszechii*; UV, B, G, R | Woodland | 0% | 0% | 100% |
| *Ch. mniszechii*; B, G, R | Woodland | 10% | 95% | 100% |
| *Ch. rajah*; UV, B, G, R | Open/Cloudy | 5% | 5% | 100% |
| *Ch. rajah*; B, G, R | Open/Cloudy | 5% | 70% | 100% |
| *Ch. rajah*; UV, B, G, R | Woodland | 5% | 5% | 100% |
| *Ch. rajah*; B, G, R | Woodland | 15% | 95% | 100% |
| *C. undatus*; UV1, UV2, B, G, Gb, R | Open/Cloudy | 5% | 20% | 100% |
| *C. undatus*; B, G, R | Open/Cloudy | 0% | 30% | 100% |
| *C. undatus*; B, Gb, R | Open/Cloudy | 10% | 55% | 100% |
| *C. undatus*; UV1, UV2, B, G, Gb, R | Woodland | 0% | 15% | 100% |
| *C. undatus*; B, G, R | Woodland | 10% | 55% | 100% |
| *C. undatus*; B, Gb, R | Woodland | 10% | 65% | 100% |

**Table S5.** Relationships between *A. planipennis* catches recorded by Francese et al. (2010), and the Q_G_/(Q_B_+Q_R_) metric calculated using sensitivity functions for *Ch. mniszechii*, *Ch. rajah*, and *C. undatus*. For *C. undatus*, the metric was calculated using both the green (G) and broadband green (Gb) photoreceptor sensitivity functions.

| Catches | Model parameter | *Ch. mniszechii* | *Ch. rajah* | *C. undatus* (G) | *C. undatus* (Gb) |
| --- | --- | --- | --- | --- | --- |
| Male | Intercept | -0.594±0.626 | 0.108±0.550 | 0.244±0.466 | -0.348±0.548 |
|  | *Z (p)* | *-0.948 (0.343)* | *0.196 (0.845)* | *0.523 (0.601)* | *-0.635 (0.525)* |
|  | Slope (index) | 6.799±1.182 | 5.731±0.998 | 5.180±0.847 | 6.549±1.074 |
|  | *Z (p)* | *5.753 (****<0.001****)* | *5.745 (****<0.001****)* | *6.115 (****<0.001****)* | *6.097 (****<0.001****)* |
|  | AIC | **49.5** | **49.6** | 48.1 | 48.5 |
|  | Intercept | 3.343±2.533 | 2.909±1.858 | 2.759±1.153 | 3.195±1.404 |
|  | *Z (p)* | *1.320 (0.187)* | *1.566 (0.117)* | *2.392 (****0.017****)* | *2.276 (****0.023****)* |
|  | Slope (index) | -11.062±11.219 | -8.325±8.355 | -7.698±5.545 | -11.221±6.715 |
|  | *Z (p)* | *-0.986 (0.324)* | *-0.997 (0.319)* | *-1.388 (0.165)* | *-1.671 (0.095)* |
|  | Slope (index^2) | 18.557±11.601 | 14.476±8.551 | 13.787±5.890 | 19.754±7.430 |
|  | *Z (p)* | *1.600 (0.110)* | *1.693 (0.091)* | *2.341 (0.019)* | *2.659 (****0.008****)* |
|  | AIC | 49.1 | 48.9 | **45.3** | **44.3** |
| Female | Intercept | 0.797±0.555 | 1.107±0.498 | 1.308±0.439 | 0.966±0.515 |
|  | *Z (p)* | *1.435 (0.151)* | *2.221 (****0.026****)* | *2.982 (****0.003****)* | *1.876 (0.061)* |
|  | Slope (index) | 3.961±1.014 | 3.285±0.868 | 2.967±0.761 | 3.759±0.975 |
|  | *Z (p)* | *3.908 (****<0.001****)* | *3.783 (****<0.001****)* | *3.897 (****<0.001****)* | *3.855 (****<0.001****)* |
|  | AIC | 43.4 | 44.0 | 43.5 | 43.7 |
|  | Intercept | 5.762±1.980 | 4.745±1.497 | 3.909±0.986 | 3.741±1.286 |
|  | *Z (p)* | *2.911 (****0.004****)* | *3.171 (****0.002****)* | *3.966 (****<0.001****)* | *2.909 (****0.004****)* |
|  | Slope (index) | -18.360±8.661 | -13.490±6.621 | -10.147±4.613 | -10.008±6.029 |
|  | *Z (p)* | *-2.120 (****0.034****)* | *-2.038 (****0.042****)* | *-2.200 (****0.028****)* | *-1.660 (0.097)* |
|  | Slope (index^2) | 23.022±8.893 | 17.138±6.725 | 13.899±4.848 | 15.181±6.601 |
|  | *Z (p)* | *2.589 (****0.010****)* | *2.548 (****0.011****)* | *2.867 (****0.004****)* | *2.300 (****0.021****)* |
|  | AIC | **39.8** | **40.5** | **38.7** | **41.0** |

**References**

Endler, J. A. (1993). The color of light in forests and Its implications. *Ecological Monographs*, *63*(1), 1-27.

Francese, J. A., Crook, D. J., Fraser, I., Lance, D. R., Sawyer, A. J., & Mastro, V. C. (2010). Optimization of trap color for emerald ash borer (Coleoptera: Buprestidae). *Forest Entomology*, *103*(4), 1235-1241.

Santer, R. D., Akanyeti, O., Endler, J. A., Galván, I., & Okal, M. N. (2023). Why are biting flies attracted to blue objects? *Proc. R. Soc. B*, *290*, 20230463.
